# Metabolism-Mediated Thrombotic Microangiopathy in an Older Patient Without Malnutrition

**DOI:** 10.7759/cureus.34951

**Published:** 2023-02-13

**Authors:** Wataru Shiraishi, Riko Okada, Yudai Tanaka, Chiaki Sano, Ryuichi Ohta

**Affiliations:** 1 Family Medicine, Shimane University Medical School, Izumo, JPN; 2 Community Care, Unnan City Hospital, Unnan, JPN; 3 Community Medicine Management, Shimane University Faculty of Medicine, Izumo, JPN

**Keywords:** general medicine, rural hospital, metabolism-mediated, thrombotic microangiopathy, older patients, vitamin b12 deficiency

## Abstract

Vitamin B12 deficiency can cause thrombotic microangiopathy (TMA)-like symptoms such as purpura caused by platelet reduction, general fatigue caused by anemia, and renal and hepatic abnormalities caused by malnutrition. TMA-like symptoms are known as metabolism-mediated TMA (MM-TMA). In MM-TMA, blood cell production is altered, and both pancytopenia and schistocytes appear. The initial presentation of MM-TMA makes it challenging to distinguish between primary and secondary TMA when patients do not present risk factors for malnutrition. We encountered an older female patient with a chief complaint of unconsciousness and loss of appetite for two days. Laboratory tests revealed pancytopenia with schistocytes. Moreover, the laboratory data revealed low serum levels of vitamin B12, indicating MM-TMA. The patient was successfully treated with intravenous vitamin B12 supplementation and discharged home. The patient had atrophic gastritis, which could have impeded the absorption of vitamin B12 from food. Among older patients without prolonged appetite loss, TMA-like symptoms should be investigated as MM-TMA induced by vitamin B12 deficiency, and prompt initiation of appropriate treatment is essential to differentiate between MM-TMA and true TMA.

## Introduction

Thrombotic microangiopathy (TMA) is a condition in which thrombi formed in microvessels cause various organ disorders [1]. Clinical manifestations are caused by thrombocytopenia and hemolysis. The three major symptoms of TMA are microangiopathic hemolytic anemia, thrombocytopenia, and organ disorders [1]. Etiologies of TMA include primary causes such as thrombotic thrombocytopenic purpura (TTP) and hemolytic uremic syndrome (HUS) caused by decreased ADAMTS13 activity, and secondary causes such as collagen disease, glomerulonephritis, or malignancy [2]. Primary TMA can be severe enough to require a plasma exchange.

In contrast, vitamin B12 deficiency and metabolism-mediated TMA (MM-TMA) are also known to cause TMA-like symptoms that can be treated with vitamin B12 supplementation [3]. Most cases occur in patients with advanced malnutrition and do not occur acutely in those with a good nutritional status [4]. This report presents a case of MM-TMA in a 96-year-old female patient accompanied by several days of unconsciousness and appetite loss. This case underscores the possibility of MM-TMA presenting with an acute course in older patients with good nutritional status. Further, this case indicates it is important to evaluate the clinical course to diagnose acute-onset MM-TMA carefully and accurately.

## Case presentation

A 96-year-old female presented to the community hospital emergency department with a chief complaint of fever and decreased oxygenation measured by home care nurses that had persisted for approximately a month. The patient had a low-grade fever of 37.2°C for one month but had no other symptoms. Therefore, the patient was followed for observation. Two days before the visit, the patient experienced symptoms of dizziness until she could not stay awake. Although her food intake decreased, she could eat and had a soft stool. On the day of the admission, the patient had an acute onset of low-grade fever (37.5°C), and SPO2 dropped to 83%-85%. The patient began to feel shortness of breath. Consequently, the patient was admitted to our hospital. Her medical history included hypertension, iron deficiency anemia/malabsorption, irritable bowel syndrome, isolated esophageal varices, and Alzheimer's disease dementia. Her medication history included rabeprazole of 10 mg/day, aspirin of 100 mg/day, furosemide of 10 mg/day, ferrous citrate of 50 mg/day, and magnesium oxide of 330 mg/day. She did not have any surgical history.

The vital signs at the time of admission were as follows: temperature, 38.2°C; heart rate, 95 beats/min; blood pressure, 151/78 mmHg; respiratory rate, 24 breaths/min; oxygen saturation, 88%-90% (room air); and normal consciousness. Physical examination revealed engorgement of the external jugular vein, pulsation of the internal cervical vein, tenderness on the right side of the abdomen, liver pain, and petechial hemorrhage on the skin. There were no obvious findings of gastrointestinal bleeding, such as hematochezia or hematemesis. Blood gas analysis showed PCO2 of 34.6 mmHg (references: 35 to 45 mmHg) and PO2 of 63.0 mmHg (references: >60 mmHg), indicating type 1 respiratory failure. A complete blood cell count showed a decreased platelet count. Furthermore, hemoglobin (Hb) and vitamin B12 levels were low, and an extremely high mean corpuscular volume (MCV) indicated macrocytic anemia. Hemolysis was suspected based on elevated levels of indirect bilirubin and lactate dehydrogenase (LDH) (Table 1).

**Table 1:** Initial laboratory data of the patient PT: prothrombin time; INR: international normalized ratio; APTT: activated partial thromboplastin time; eGFR: estimated glomerular filtration rate; ADAMTS: a disintegrin and metalloproteinase with thrombospondin motifs; SARS-CoV-2: severe acute respiratory syndrome coronavirus 2

Parameters	Level	Reference
White blood cells	3.60	3.5–9.1×10^3^/μL
Neutrophils	88.4	44.0–72.0%
Lymphocytes	8.8	18.0–59.0%
Monocytes	2.5	0.0–12.0%
Eosinophils	0.1	0.0–10.0%
Basophils	0.2	0.0–3.0%
Red blood cells	1.30	3.76–5.50×10^6^/μL
Hemoglobin	6.0	11.3–15.2 g/dL
Hematocrit	18.4	33.4–44.9%
Mean corpuscular volume	141.4	79.0–100.0 fL
Platelets	9.7	13.0–36.9×10^4^/μL
Total protein	6.2	6.5–8.3 g/dL
Albumin	3.7	3.8–5.3 g/dL
Total bilirubin	2.8	0.2–1.2 mg/dL
Direct bilirubin	0.8	0.0–0.4 mg/dL
Aspartate aminotransferase	46	8–38 IU/L
Alanine aminotransferase	17	4–43 IU/L
γ-Glutamyl transpeptidase	10	<48 IU/L
Lactate dehydrogenase	1209	121–245 U/L
Blood urea nitrogen	35.8	8–20 mg/dL
Creatinine	0.75	0.40–1.10 mg/dL
Estimated glomerular filtration rate	52.9	>60.0 mL/min/L
Serum sodium	140	135–150 mEq/L
Serum potassium	4.1	3.5–5.3 mEq/L
Serum chloride	108	98–110 mEq/L
Serum calcium	8.5	8.8–10.2 mg/dL
Serum phosphorus	4.1	2.7–4.6 mg/dL
Serum magnesium	2.3	1.8–2.3 mg/dL
Serum glucose	138	70–110 mg/dL
Creatinine kinase	43	56–244 U/L
C-reactive protein	0.07	<0.30 mg/dL
Vitamin B12	<148	187-883 pg/mL
Erythrocyte sedimentation rate	16	2–10 mm
PT	83.3	70–130%
PT-INR	1.10	
APTT	26.5	25–40 s
Fibrinogen degradation products	5.4	<5 μg/mL
SARS-CoV-2	Negative	Negative
Urine test		
Leukocyte	(3+)	(–)
Nitrite	(+)	(–)
Protein	(2+)	(–)
Glucose	(-)	(–)
Urobilinogen	(1+)	(–)
Bilirubin	(–)	(–)
Ketone	(–)	(–)
Blood	(3+)	(–)

**Figure 1:**
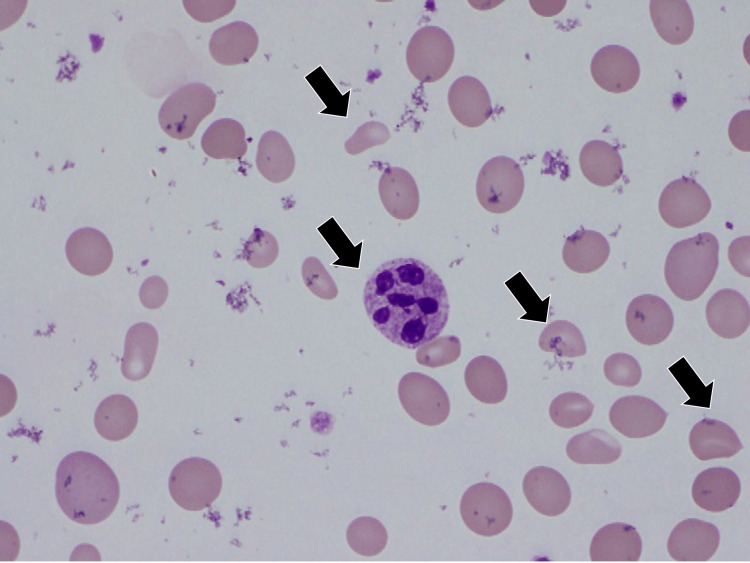
A blood smear showing schistocytes and a hyper-segmented neutrophil (black arrows)

Crushed red blood cells were observed in peripheral blood smears (Figure 1).

Urinalysis revealed leukocytes (3+), protein (2+), occult blood (3+), and bacteria. TMA was suspected due to crushed erythrocytes, macrocytic anemia, low platelet count, increased LDH and elevated indirect bilirubin levels. In this case, HUS was suspected because of the low Hb level and the presence of crushed red blood cells in the peripheral hemogram. However, there were no petechiae or preceding bloody diarrhea and no decrease in the estimated glomerular filtration rate or increase in creatinine levels, which could lead to the diagnosis of acute kidney injury. We did not actively suspect collagen disease or vasculitis based on an erythrocyte sedimentation rate of 16 mm (baseline, 3-15) and a C-reactive protein level of 0.07 mg/dL (baseline, <0.30).

We suspected MM-TMA due to vitamin B12 deficiency and extremely high MCV and LDH. TTP could not be excluded; however, the patient’s vital signs were stable. We tested ADAMTS13's activity and administered supplemental fluids, blood transfusions, and intravenous vitamin B12 replacements. Three days later, ADAMTS13 inhibitor results were negative. The upper gastroscopy revealed atrophic gastritis without ulceration or malignancy. The patient’s condition drastically improved with intravenous vitamin B12 supplementation. The patient’s laboratory data also improved, with a Hb level of 11 g/dL on the 12^th^ day of admission. She was discharged home on the 18^th^ day of admission. In the outpatient department, she was controlled with a tablet of vitamin B12 without anemia.

## Discussion

This case report suggests that MM-TMA can appear in older patients with normal nutritional status and can be characterized by extremely high levels of MCV and LDH with no significant changes in vital signs. Regarding vitamin B12 metabolism, 20 genes may influence cobalamin absorption, storage, and intracellular processing [5]. Thus, cobalamin deficiency may occur due to gene mutation or other conditions that affect absorption, transport, and storage. Cobalamin is stored in the liver; therefore, cobalamin deficiency may not appear for 3-6 years, even with decreased intake and absorption [6,7]. Thus, vitamin B12 deficiency may progress gradually. In the present case, the patient was older; therefore, hereditary factors may not have been possible. Previous studies have shown that aging-related atrophic gastritis and intestinal atrophy due to aging can cause vitamin B12 deficiency in frail older individuals [8,9]. Although vitamin B12 deficiency is rare in elderly patients with appropriate nutritional conditions, aging and atrophy of the gastrointestinal tract can induce vitamin B12 deficiency. 

Thus, vitamin B12 deficiency can cause hematological abnormalities such as erythroblastic anemia and MM-TMA [3]. Cobalamin deficiency can lead to hyperhomocysteinemia and methylmalonic aciduria, leading to the generation of reactive oxygen species, which results in endothelial dysfunction, platelet activation, increased tissue factor expression, and subsequent activation of the coagulation cascade, leading to a hematological condition of TMA [4]. The difference between MM-TMA and other critical TMA can be determined based on changes in vital signs and the speed of clinical progression [3]. Based on its pathophysiology, MM-TMA can progress gradually with abnormal metabolism during hematogenesis [3]. Thus, general physicians should include MM-TMA in differential diagnoses of patients presenting the gradual progression of unconsciousness and vital signs. 

Herein, we describe a female patient with MM-TM aged 96 years, which is not an epidemiologically favorable age for the disease. Furthermore, there was no evidence of cognitive dysfunction, ataxia, or neuropsychiatric symptoms in this patient, making a diagnosis of cobalamin metabolism disorder unlikely. Hemolytic anemia (the appearance of crushed red blood cells) decreases red blood cells and platelets, and renal dysfunction usually rushes physicians to investigate TTP and HUS. However, in this case, the vitamin B12 level was < 148 pg/mL at the emergency room visit. In rural contexts, many older patients with unconsciousness are admitted to rural community hospitals. General physicians must approach them comprehensively and systematically [10,11]. In their investigation, anemia and blood smears were checked to rule out vitamin B12 deficiency complicated by MM-TMA.

## Conclusions

Vitamin B12 deficiency and abnormal cobalamin metabolism can cause MM-TMA in older patients with loss of appetite. The prevalence of vitamin B12 may change among older patients because of gastrointestinal tract atrophy. Especially in rural contexts with an increased number of older patients, general physicians should investigate the presence of vitamin B12 deficiency complicated by MM-TMA, accompanied by extremely high levels of MCV and LDH.
